# Service integration: opportunities to expand access to antiretroviral therapy for people who inject drugs in Tanzania

**DOI:** 10.7448/IAS.18.1.19936

**Published:** 2015-07-21

**Authors:** Barrot H Lambdin, Jessie K Mbwambo, Robert M Josiah, Robert D Bruce

**Affiliations:** 1 Behavioral and Urban Health Program, RTI International, San Francisco, CA, USA; 2 Department of Epidemiology and Biostatistics, University of California, San Francisco, CA, USA; 3 Department of Global Health, University of Washington, Seattle, WA, USA; 4 Department of Psychiatry, Muhimbili University of Health and Allied Sciences, Dar es Salaam, Tanzania; 5 National AIDS Control Program, Ministry of Health and Social Welfare, Dar es Salaam, Tanzania; 6 Department of Medicine, Cornell Scott-Hill Health Center, New Haven, CT, USA; 7 School of Medicine, Yale University, New Haven, USA

**Keywords:** HIV/AIDS, antiretroviral therapy, methadone, people who inject drugs, service integration, implementation science

Research highlighting both individual and public health benefits of expanded access to antiretroviral therapy (ART) has stimulated efforts to increase the number of people accessing ART. Recent evidence suggests that moving towards earlier ART initiation is associated with improved clinical outcomes, survival and HIV prevention benefits, including reduced incidence of HIV infection at the community level [1–3]. This evidence highlights the collective importance for early detection of HIV, prompt linkage to care and early initiation of ART to capitalize on the treatment and prevention benefits [4,5].

Despite the benefits of ART access, HIV-positive people who inject drugs (PWID) are less likely to receive ART than people who do not inject drugs. PWID consistently face barriers which limit the availability and accessibility of HIV prevention and treatment interventions [6]. Where programmes exist, many fail to reach those who could benefit due to requirements that make it difficult for patients to enter and remain in services [7]. Structural barriers, such as punitive laws, uncoordinated services, programmes with burdensome requirements, lack of transportation, high costs, or fear of stigmatization, impact utilization of medical services by PWID. In addition, physicians have delayed or withheld treatment for fear of on-going drug use, risky behaviours and non-adherence potentially leading to development of drug resistance [8,9], and as a result, only 4 per 100 HIV-positive PWID worldwide receive ART [10].

Since the late 1990s, heroin injection has become widespread in Dar es Salaam, Tanzania, and is spreading throughout the region [11,12]. The current estimates on mainland Tanzania suggest 30,000 PWID with an HIV prevalence of 36% [13,14]. Similar to many other countries, the impact of structural barriers including punitive laws, law enforcement abuses, discrimination and uncoordinated services have also been documented in Tanzania [15]. These barriers are important determinants of HIV vulnerability and must be addressed to reduce HIV risk, transmission, barriers to services and community viral load among PWID [8,16]. These issues are critically important in their own right, and especially given the role PWID play as a “bridge” between drug using and non-drug using populations.

Tanzania responded to the HIV epidemic among PWID by implementing the first publicly funded methadone assisted therapy programme on the mainland of sub-Saharan Africa, beginning in 2011. To date, over 2,500 clients have enrolled into methadone among three clinics in Dar es Salaam and one in Zanzibar. Recent research showed that patient retention within Tanzania's programme was comparable to programmes in North America, Europe and Asia [17].

As part of routine care, methadone clients were offered HIV counselling and testing at enrolment into methadone and every six months thereafter. If a client was HIV antibody positive, a blood sample was taken and transported to the central laboratory for processing and the patient was then referred to care and treatment centres for HIV clinical services. With this model, a recent analysis highlighted that 41% of ART-eligible methadone clients initiated ART within 90 days of their HIV-positive test result [18]. These results indicate some success in effectively linking PWID to ART. However, given that clients in Tanzania currently present to the clinic on a daily basis for their methadone dose, structural improvements are possible. Further programmatic initiatives must prioritize the design and evaluation of implementation strategies that properly integrate the full range of ART services with methadone.

While a community engagement process is essential to develop an integrated approach for care delivery [19], some key elements that can be critically important for methadone clinics in Tanzania include:

- **Health workforce**. Integrating services into one location for clients is a necessary but insufficient structural solution, as simply adding more responsibilities to an already overburdened workforce will not yield the results needed. The linchpin for effective integration of HIV care will be to ensure an adequate number of health workers with the right skillset [20]. By building a strong workforce at the methadone clinics, providers will be able to effectively attend to the needs of their clients, efficiently offering all of the necessary clinical, laboratory and pharmacy services in one location.
- **
Point-of-care (POC) diagnostics**. As recent research has highlighted the feasibility of POC CD4 diagnostics in programmatic settings [21,22], new POC technologies have the ability to revolutionize the structure of service delivery by allowing for eligibility determination and ART initiation on the same day and in the same place as an HIV diagnosis. Implementation of POC CD4 diagnostics within methadone clinics will streamline laboratory procedures, allowing providers to offer real-time information to their clients with regard to their eligibility for ART.
- **Alert and reminder systems for providers**. Alert and reminder systems make up the majority of health information technology interventions that address the HIV treatment cascade [23]. In the context of a chaotic clinical environment where multiple mental and physical health conditions are being managed at any given time, these tools are pivotal to assist providers in the timely completion of tasks for successful ART initiation and retention.

To put it simply, the current state of affairs for PWIDs accessing services is unacceptable. We, as a global HIV community, have an ethical imperative to ensure access to key HIV prevention and treatment services for PWID, just as we would for any other community. And, one of the prominent barriers to improve access remains the structural obstacles that PWID face. Failing to address these will hamper efforts to realize an AIDS-free generation.

The Government of Tanzania has demonstrated tremendous leadership by embracing methadone as a key medical intervention for PWID, but the remaining structural barriers that hamper access to key medical services must be addressed. Currently, methadone delivery requires frequent clinic visits for clients, providing the perfect opportunity to address a number of clinical conditions. In addition to further scaling-up methadone services, well-designed and sufficiently staffed integration of ART into methadone services, using a combination of strategies that have been successful in other settings, is critical for us to build a patient-centred model of care to maximize HIV prevention and treatment benefits for PWID. In addition, further initiatives must address law enforcement abuse as well as the stigma and discrimination faced by PWID. These efforts will be critical to improve the effectiveness and efficiency of the global AIDS response.
